# Clinical significance of late gadolinium enhancement in pediatric patients with hypertrophic cardiomyopathy

**DOI:** 10.1186/1532-429X-15-S1-O97

**Published:** 2013-01-30

**Authors:** Brandon M Smith, Adam L Dorfman, Sunkyung Yu, Prachi Agarwal, Maryam Ghadimi Mahani, Jimmy C Lu

**Affiliations:** 1 University of Michigan Congenital Heart Center, University of Michigan, Ann Arbor, MI, USA

## Background

Late gadolinium enhancement (LGE) is associated with adverse events in adults with hypertrophic cardiomyopathy (HCM). However, the extent and clinical significance of LGE in the pediatric population has not been well described. This study sought to characterize the pattern of LGE and its relation to hypertrophy, strain, and clinical outcomes in pediatric patients with HCM.

## Methods

This single center, retrospective study included all patients 21 years or less with a clinical diagnosis of HCM who underwent cardiac magnetic resonance (CMR) from 2007-2012. Using a 16-segment model, segments with hypertrophy or LGE were identified by agreement of two experienced readers blinded to outcome. Circumferential and radial strain were evaluated by segment, using feature tracking software (TomTec, Unterschleissheim, Germany). The composite outcome was defined as death, non-sustained ventricular tachycardia (VT), or ventricular fibrillation (VF).

## Results

Thirty patients (mean age 14.1±3.2 years) were included, with 11 patients (37%) confirmed by genotype. LGE was present in 17 patients (57%), predominantly in the basal and mid-ventricular septum and anterior wall, all in a mid-myocardial pattern, with a median of 3 segments per patient (interquartile range 2-5). LGE was closely related to hypertrophy. Patients with LGE had an odds ratio (OR) of 38.6 for hypertrophy (p<0.001); no LGE was detected in patients without hypertrophy. Segments with LGE had decreased radial strain (basal segments 20.7 vs. 70.9%, p=0.01; mid segments 32.1 vs. 62.9%, p=0.0003) and circumferential strain (basal segments -23.2 vs. -29.3%, p=0.04; mid segments -26.1 vs. -29.4%, p=0.09). Decreased strain may be independent of hypertrophy (radial strain 29.0 vs. 44.7% in segments with hypertrophy but no LGE, p=0.07). After median follow-up of 12.5 months (interquartile range 4-36), 7 patients had a negative outcome, with a strong trend toward increased odds of death, VT, or VF in patients with LGE (OR 6.2, p=0.1). Patients with an event had more segments of LGE than patients without an event (median 4 (interquartile range 2-7) vs. 0 (interquartile range 0-2), p=0.01). One episode of VT occurred in a patient without LGE.

## Conclusions

In pediatric patients with HCM, LGE is unlikely to occur in the absence of hypertrophy. Similar to the adult HCM population, myocardial deformation is reduced in areas with LGE. LGE may relate to negative outcomes in a pediatric population, although further study is necessary to confirm this relationship in a larger population, not pre-selected for clinical need of CMR.

## Funding

None.

